# Shared and unique lifetime stressor characteristics and network connectivity predict adolescent anxiety and depression

**DOI:** 10.1101/2024.10.25.620373

**Published:** 2024-12-01

**Authors:** Yueyue Lydia Qu, Sidhant Chopra, Shijie Qu, Carrisa V. Cocuzza, Loïc Labache, Clemens C.C. Bauer, Francesca Morfini, Susan Whitfield-Gabrieli, George M. Slavich, Jutta Joormann, Avram J. Holmes

**Affiliations:** 1 Department of Psychology, Yale University, New Haven, CT, USA; 2 Wu Tsai Institute, Yale University, New Haven, CT, USA; 3 Orygen, Melbourne, VIC, Australia; 4 Centre for Youth Mental Health, The University of Melbourne, Melbourne, VIC, Australia; 5 Department of Psychiatry, Brain Health Institute, Rutgers University, Piscataway, NJ, USA; 6 Department of Psychology, Northeastern University, Boston, MA, USA; 7 Center for Cognitive & Brain Health, Northeastern University, Boston, MA, USA; 8 Department of Brain and Cognitive Sciences and McGovern Institute for Brain Research, Massachusetts Institute of Technology, Cambridge, MA, USA; 9 Department of Psychiatry and Biobehavioral Sciences, University of California, Los Angeles, CA, USA

**Keywords:** Major life stressor characteristics, resting-state functional networks, longitudinal prediction, anxiety, depression, adolescence

## Abstract

**Background:**

Exposure to major life stressors and aberrant functional connectivity have been linked to anxiety and depression, especially during adolescence. However, whether specific characteristics of life stressors and functional network connectivity act together to differentially predict anxiety and depression symptoms remains unclear.

**Methods:**

We utilized baseline lifetime stressor exposure and resting-state functional magnetic resonance imaging data in a longitudinal sample of 107 adolescents enriched for anxiety and depressive disorders. We examined five stressor characteristics: physical danger, interpersonal loss, humiliation, entrapment, and role change/disruption. Anxiety and depression symptoms were assessed at baseline, 6-month and 12-month follow-ups. Linear mixed-effect models tested if lifetime severity of these stressor characteristics, functional connectivity within and between frontoparietal, default, and ventral attention networks, and their interactions differentially predicted anxiety and depression symptoms at two 6-month follow-ups.

**Results:**

Greater lifetime severity of physical danger and humiliation predicted higher anxiety symptoms. Greater lifetime entrapment severity predicted higher anxiety and depression symptoms. After including within- and between-network functional connectivity and other predictive characteristics, only the effects of lifetime entrapment severity remained significant. Lifetime entrapment severity more strongly predicted anxiety symptoms in youth with higher default network connectivity. Greater functional connectivity between frontoparietal and default networks predicted increased depression symptoms.

**Conclusions:**

Our study is the first to use lifetime severity of distinct stressor characteristics and resting-state functional connectivity jointly to predict adolescent anxiety and depression symptoms. These results imply certain stressor characteristics and functional connectivity metrics as specific predictors of anxiety or depression and highlight entrapment as a shared predictor for anxiety and depression.

## Introduction

Exposure to major life stressors is a strong risk factor for the onset and subsequent recurrence of affective disorders (1–5), especially in adolescence when there is increased brain plasticity and heightened vulnerability to the emergence of psychopathology (6–10). However, stressors come in many different forms and are diverse with respect to both their characteristics and associations with mental health outcomes (11–13). Results from large-scale, prospective cohort studies have suggested that certain characteristics of major life stressors may preferentially increase risk for specific clinical outcomes. For instance, stressors that involve devaluation of the self, such as interpersonal loss and humiliation, are theorized to preferentially heighten risk for depression (14–18). In contrast, stressors marked by a threat to one’s physical integrity, such as danger, are theorized to be stronger predictors of anxiety (14,16,17,19). Stressors characterized by feelings of failure without any means of escape, such as entrapment, predict both anxiety and depression (14,20). These patterns may be explained by the cognitive content-specificity hypothesis of anxiety and depression which posits that anxiety and depression can be discriminated by distinct forms of negative beliefs (21) and which has been partially supported (22,23). However, the potential neural mechanisms linking distinct stressor characteristics with adolescent anxiety and depression remain unknown.

Major life stressors can induce long-lasting effects on neurobiological functioning (24–28), particularly when they occur during childhood and adolescence (25,26,29–31). Prior neuroimaging studies have associated changes in brain functioning during monetary reward and emotional face tasks with exposure to life stressors (32–35). Altered brain functioning is also evident when considering resting-state functional connectivity (RSFC) patterns within and between the frontoparietal (36), default (37,38), and salience/ventral attention (39) networks among people who were exposed to early-life stress and trauma (29,40–43). Because altered connectivity within the frontoparietal, default and ventral attention networks are theorized to underlie dysfunctional cognitive (44–46), self-referential (45–47) and salience processing (45,48) respectively in anxiety and depression, it is possible that disruptions within and between these functional networks moderate the process of stress exposure leading to psychopathology. Although prior research has examined the extent to which intrinsic patterns of functional connectivity predict adolescent anxiety and depression following general stress exposure (49,50), these studies have not considered the interactions between neurobiological factors and life experience in differentiating anxiety and depression. Longitudinal associations linking distinct dimensions of life stressor exposure, network connectivity, and clinical symptoms thus remain to be established.

### Present Study

To address these gaps in knowledge, we first conducted hypothesis-driven analyses investigating if distinct characteristics of major life stressors occurring over the entire life course differentially predicted anxiety and depression symptoms at 6-month and 12-month follow-ups. We then explored if total lifetime severity of any stressor characteristics, RSFC metrics within and between the frontoparietal, default, and ventral attention networks, and their interactions predicted anxiety and depression symptoms at both follow-ups. These analyses were conducted in a longitudinal sample of adolescents recruited from school-based and hospital-based child treatment programs (51). Most participants had a current diagnosis of at least one anxiety or depressive disorder at the time of baseline assessment. Based on the prior research summarized above, we tested three hypotheses using linear mixed-effects (LME) models: (a) greater lifetime severity of physical danger at baseline would differentially predict higher anxiety symptoms at both follow-ups; (b) greater lifetime severity of interpersonal loss and humiliation at baseline would differentially predict higher depression symptoms at both follow-ups; (c) greater lifetime severity of entrapment at baseline would predict higher levels of both depression and anxiety symptoms at both follow-ups. In further exploratory analyses, we assessed whether any stressor characteristics and RSFC metrics within and between the three large-scale networks predict anxiety/depression symptoms at two 6-month follow-ups, and whether these RSFC metrics would moderate the strengths of prospective associations between stressor characteristics and anxiety/depression symptoms.

## Methods and Materials

### Participants

**Figure 1.**
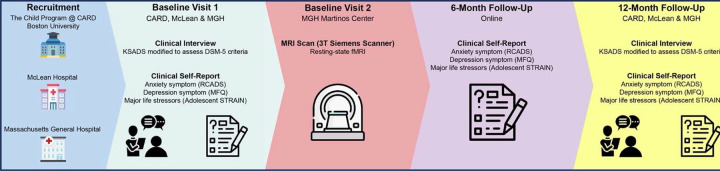
Schematic representation of the Boston Adolescent Neuroimaging of Depression and Anxiety (BANDA) study. Participants were recruited across three sites and underwent four study sessions, including in-person clinician evaluations, self-report measures, neuroimaging, and online and in-person follow-up assessments. The two clinical and imaging baseline visits occurred within two weeks from each other. Participants completed an online batter of self-report questionnaires 6 months after their second baseline visit. Finally, they went through on-site clinical evaluations and completed an additional batter of self-report questionnaires 12 months after their second baseline visit. Picture icons were downloaded from www.freepik.com.

Data were collected from 215 adolescents (*M*_age_ = 15.44, range = 14–17 years old) enrolled in the Boston Adolescent Neuroimaging of Depression and Anxiety (BANDA) study (51) and assessed at 6-month intervals after the initial visit for up to 12 months. The present analyses were restricted to data from the baseline, 6-month follow-up and 12-month follow-up assessments. Resting-state functional magnetic resonance imaging (rsfMRI) data was available for 203 participants at baseline (51). Out of these 203 adolescents, 107 had available self-reported anxiety and depression symptoms at baseline, 6- and 12- month follow-up assessments and were included in the final analytical sample (Table 1; Figure 1). Of these 107 participants, 62% (*n* = 66) had a current diagnosis of at least one anxiety or depressive disorder. These diagnoses were given by a blinded, licensed clinical psychologist based on the Diagnostic and Statistical Manual of Mental Health Disorders, 5^th^ Edition (DSM-5; 51) and reached moderate to substantial inter-rater agreement (51).

### Measures of social-psychological characteristics of life stressors

Exposure to acute and chronic stressors occurring over the entire life course was assessed using the Stress and Adversity Inventory for Adolescents (STRAIN) (53). The STRAIN measured the total lifetime severity for each social-psychological stressor characteristic, including physical danger, interpersonal loss, humiliation, entrapment, and role change/disruption, for each participant based on their self-reported acute and chronic stressors (see https://www.strainsetup.com). Examples of stressors linked to each of these five characteristics have been described elsewhere (53). Total lifetime severity scores for these five characteristics were used to predict the levels of anxiety and depression symptoms at two six-month follow-up assessments. Brief definitions for these stressor characteristics are as follows (14,17,54,55):

**Physical danger**. The degree of potential future threat to one’s physical safety that might occur as a result of the stressor.
**Interpersonal loss**. Diminution of a sense of connectedness or well-being as a result of a real or realistically imagined loss of a person by death or by separation.
**Humiliation**. The likelihood of a stressor rendering a person devalued in relation to others or self, usually due to rejection or a sense of core failure.
**Entrapment**. Ongoing circumstances of marked difficulty of at least 6 months’ duration that the individual can reasonably expect to persist or get worse, with little or no possibility that a resolution can be achieved as a result of anything that might reasonably be done.
**Role change/disruption**. Life transitions that involve addition, subtraction or change of social roles.

The STRAIN has demonstrated excellent test-retest reliability, good concurrent and discriminant validity, as well as predictive utility in relation to various clinical outcomes including anxiety and depression (53,56).

### Measures of anxiety and depression symptoms

Self-reported anxiety symptoms in this study were assessed by the Revised Children’s Anxiety and Depression Scale (RCADS) (57). The RCADS has exhibited excellent internal consistency, test-retest reliability, convergent and discriminant validity (57). The RCADS have six subscales: separation anxiety disorder, social phobia, generalized anxiety, panic disorder, obsessive-compulsive disorder and low mood. Total anxiety symptoms for each participant were computed by summing the four anxiety subscales (Separation Anxiety Disorder, Social Phobia, Generalized Anxiety and Panic Disorder).

Self-reported depression symptoms were assessed by the Mood and Feelings Questionnaire (MFQ) (58,59). Prior studies have found the MFQ to be a reliable and valid measure of adolescent depression in both clinical and non-clinical samples across different populations (60–63). We used the total MFQ score as the measure of depression symptoms for each participant.

### Neuroimaging

#### Data acquisition and processing

Functional and anatomical neuroimaging data were acquired at baseline assessment using a 3-Tesla Siemens Prisma scanner with a 2D multi-band gradient-recalled echo-planar imaging (EPI) sequence. Each participant underwent four 5.8-minute resting-state functional MRI (rsfMRI) runs, consisting of two runs with opposite phase encoding directions (AP/PA). Each rsfMRI scan was acquired using 2mm isotropic resolution and a TR of 800ms. Full details of the acquisition protocol can be found elsewhere (64).

The acquired rsfMRI data then went through the previously established Human Connectome Project (HCP) minimal preprocessing pipelines (65). Minimally preprocessed T1w images (65) went through bias- and distortion- correction using the *PreFreeSurfer* pipeline and registered to MNI space. Cortical surface reconstruction was conducted using FreeSurfer v5.2 using recon-all adapted for high-resolution images. The reconstructed surface meshes were then registered to the Conte69 surface template (66). During preprocessing, the fMRI data were first corrected for gradient-nonlinearity-induced distortions. The fMRI time series in each frame were then realigned to the single-band reference image to correct for subject motion using rigid body transformation (67,68) with FSL. The resulting single-band image underwent spline interpolation to correct for distortions and was then registered to the T1w image (69). The registered fMRI volumes then went through nonlinear registration to the Conte69 surface template (66) and mapped to the standard CIFTI grayordinate coordinate space. Further details about the HCP minimal preprocessing pipelines of structural and functional images can be found elsewhere (65). The minimally preprocessed fMRI data for each run were then denoised using ICA+FIX (70,71) pre-trained using HCP_hp2000.RData and aligned across participants using MSMAll multi-modal surface registration (72,73).

#### Resting-state functional connectivity

We defined 400 cortical regions of interest (ROIs) using a previously validated atlas (74). Resting-state functional connectivity (RSFC) was measured by Pearson’s *r* correlations between the mean time series of each pair of ROIs. The average FC matrix across all runs in each participant was computed after applying Fisher Z-transformation and used for subsequent analyses.

**Figure 2.**
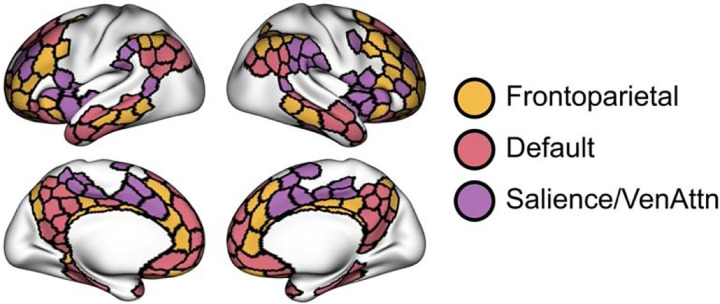
The functional network organization of the human cerebral cortex revealed through intrinsic functional connectivity. Colors reflect regions estimated to be within the same network. Cortical regions-of-interest (ROIs) defined by Schaefer’s parcellation (74) and assigned to frontoparietal (yellow), default (red), and salience/ventral attention (purple) networks (75).

Specifically, this study focused on RSFC within and between the frontoparietal, default, and ventral attention networks (Figure 2) according to the 17-network solution (75) as predictors of anxiety and depression symptoms at two 6-month follow-ups. Within-network connectivity was assessed by averaging the pairwise RSFC of all regions assigned to that network, resulting in three within-network connectivity values per individual. “Between” network connectivity was assessed by computing the pair-wise correlations of each ROI in one network (e.g., frontoparietal) to each ROI in the other network (e.g., default) and averaging across them, resulting in three between network connectivity values per individual.

### Covariates

The following covariates were dummy coded, converted to factors and entered into each LME model: participant’s race (White: Yes=1, No=0; African American: Yes=1, No=0; Asian: Yes=1, No=0; Hawaiian: Yes=1, No=0), ethnicity (Hispanic: Yes=1, No=0), sex (female=0, male=1), participant’s current diagnostic group (Depression [having a current diagnosis of at least one depressive disorder]: Yes=1, No=0; Anxiety [having a current diagnosis of at least one anxiety disorder and no depressive disorder]: Yes=1, No=0; Control [having no current or lifetime diagnosis of any psychiatric disorder]: Yes=1, No=0). We included current diagnosis of depressive and anxiety disorders as covariates because there were significant differences in depression and anxiety symptoms severity as well as total lifetime severity of stressor characteristics across the three diagnostic groups (Supplemental Result 1). Participant’s age at scan was entered as a continuous covariate in each LME model. We additionally included baseline depression symptoms in each LME model predicting anxiety symptoms and baseline anxiety symptoms in each LME model predicting depression symptoms as covariates to parse out unique predictors of each symptom (i.e., anxiety, depression) over time.

### Statistical analyses

We constructed linear mixed-effect (LME) models using the *lme4* package in Rv4.2.0 (76) with restricted maximum likelihood estimation (REML) to test our hypotheses. Each hypothesis-driven LME model assessed if total lifetime severity for each stressor social-psychological characteristic (i.e., physical danger, interpersonal loss, humiliation, entrapment) at baseline predicted each symptom (i.e., anxiety, depression) at two 6-month follow-ups. We used the *cAIC4* package *v1.0* (77) to determine whether each of these models yielded lower conditional Akaike information criterion (cAIC) when fitted with a random intercept or with a random slope plus a random intercept. We also included covariates in each model to test if the fixed effects of these stressor characteristics were robust to the inclusion of potential confounders. Continuous predictors, covariates and outcome variables were standardized to make the beta estimates more interpretable and to avoid multicollinearity.

Finally, we included the total lifetime severity of all stressor characteristics, all RSFC metrics and their interactions in a single “unified LME model” predicting each symptom (i.e., anxiety, depression) at two 6-month follow-ups. In each unified LME model, we explored if total lifetime severity of any stressor characteristics, any RSFC metrics within and between the frontoparietal, default, and ventral attention networks, and their interactions emerged as significant predictors of each symptom. Each unified LME model was fitted either with a random intercept or with a random intercept plus a random slope. Potential confounders were included in each unified LME model and all continuous variables were standardized. As an example, the formula for the unified LME model predicting prospective anxiety symptoms with a random slope and intercept was:

AnxietySx~(PhysicalDanger+InterpersonalLoss+Humiliation+Entrapment+RoleReversal)*(RSFCwithinFPN+RSFCwithinDN+RSFCwithinVAN+RSFCbetwFPN-DN+RSFCbetwFPN-VAN+RSFCbetwDN-VAN)+DepressionDx+AnxietyDx+BaselineDepressionSx+Race+Ethnicity+BaselineAge+sex+time+(1+time∣Subject).

The unified LME model predicting prospective depression symptoms with only a random intercept was:

DepressionSx~(PhysicalDanger+InterpersonalLoss+Humiliation+Entrapment+RoleReversal)*(RSFCwithinFPN+RSFCwithinDN+RSFCwithinVAN+RSFCbetwFPN-DN+RSFCbetwFPN-VAN+RSFCbetwDN-VAN)+DepressionDx+AnxietyDx+BaselineAnxietySx+Race+Ethnicity+BaselineAge+sex+time+(1∣Subject).

Since total lifetime severity scores for the five stressor characteristics were highly intercorrelated (Supplemental Table 1), as expected, the resulting unified LME models may exhibit high multicollinearity (VIF≥5) despite standardization of predictor and outcome variables. Hence, for each unified LME model, we started with including all five stressor characteristics, iteratively removing different subsets of one to four stressor characteristics, rerunning the model and recomputing cAIC. We selected the optimal subset of stressor characteristics yielding the lowest conditional Akaike information criterion (cAIC) for each unified LME model. The conditional R^2^ captured by each optimal unified LME model was determined using the *performance* package (78) in Rv4.2.0.

## Results

### Total lifetime severity of stressor characteristics differentially predicts prospective anxiety and depression symptoms

Across all hypothesis-driven LME models predicting anxiety symptoms at two 6-month follow-ups, the models fitted with random slopes plus random intercepts yielded lower cAICs than the corresponding models fitted with only random intercepts (Supplemental Table 3). On the other hand, all hypothesis-driven LME models predicting prospective depression symptoms were singular when fitted with both random slopes and random intercepts (Supplemental Table 4). Therefore, we will focus on results from LME models fitted with random slopes and intercepts when prospective anxiety symptoms severity is the predicted variable and focus on results from LME models fitted with only random intercepts when prospective depression symptoms is the predicted variable.

Consistent with our hypothesis that lifetime physical danger severity is a specific predictor of anxiety, the LME analyses revealed that greater lifetime severity of physical danger at baseline predicted higher anxiety (*β* = 0.25, *p* = 3.63×10^−5^; Supplemental Table 4) but not depression symptoms (*β* = −70.0079, *p* = 0.90; Supplemental Table 5) at follow-up, after accounting for potentially confounding covariates.

Failing to support our second hypothesis theorizing interpersonal loss as a specific predictor of depression, the second set of hypothesis-driven LME models indicated that the main effects of lifetime severity of interpersonal loss on both anxiety (*β* = 0.039, *p* = 0.55; Supplemental Table 6) and depression symptoms were not robust to the inclusion of potentially confounding covariates (*β* = 0.057, *p* = 0.29; Supplemental Table 7). These results suggest that individual differences in the lifetime severity of interpersonal loss does not predict prospective depression symptoms above and beyond anxiety symptoms and depression diagnosis at baseline.

Contrary to our third hypothesis, the third set of hypothesis-driven LME models revealed that greater lifetime severity of humiliation at baseline predicted higher anxiety (*β* = 0.31, *p* = 2.56×10^−6^; Supplemental Table 8) but not depression symptoms (*β* = 0.034, *p* = 0.62; Supplemental Table 9) at follow-up, after accounting for potentially confounding covariates. These data suggest that lifetime severity of humiliation may be a specific risk factor for anxiety rather than depression.

Finally, the fourth set of hypothesis-driven LME models revealed that greater total lifetime severity of entrapment predicted both anxiety (*β* = 0.39, *p* = 5.20×10^−8^; Supplemental Table 10) and depression symptoms at two 6-month follow-ups, after accounting for potentially confounding covariates (*β* = 0.15, *p* = 0.05; Supplemental Table 11). These results suggest that lifetime severity of entrapment may be a shared risk factor for both anxiety and depression.

### Total lifetime severity of stressor characteristics, large-scale brain networks and symptoms of anxiety and depression

Next, we sought to determine if prospective associations between stressor characteristics and clinical symptoms are moderated by patterns of functional connectivity within and between the large-scale brain networks theorized to underlie the expression of affective illness. To this end, we used two unified LME models to explore if total lifetime severity of any stressor characteristics, any RSFC metrics within and between the frontoparietal, default, and ventral attention networks, and their interactions emerged as significant predictors of prospective anxiety and depression symptoms. The unified LME model predicting prospective anxiety symptoms yielded the lowest cAIC when fitted with both a random slope and intercept and when total lifetime severity for physical danger and entrapment were entered as predictors along with all RSFC metrics (Supplemental Table 12). The optimal unified model for anxiety had a conditional R^2^ of 0.873. The unified LME model predicting prospective depression symptoms yielded the lowest cAIC when fitted with only a random intercept and when total lifetime severity entrapment was the only stressor characteristic in the model (Supplemental Table 13). The optimal unified model for depression had a conditional R^2^ of 0.759. Below, we focus on the results from the optimal unified LME models.

**Figure 3.**
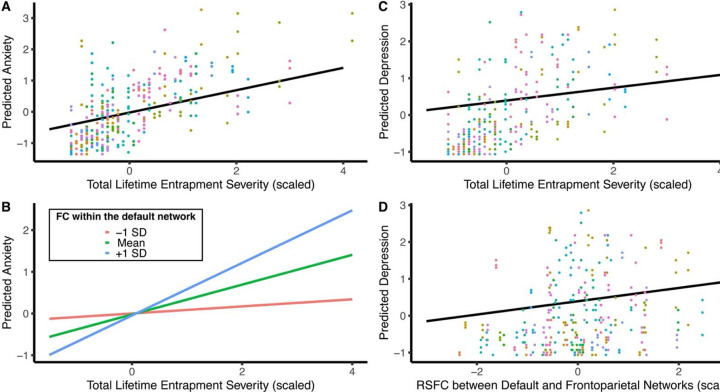
(A) The association between total lifetime severity of entrapment and the sums of marginal fit and conditional residuals from the fitted LME models predicting anxiety symptoms depression symptoms at two 6-month follow-ups; (B) The association between total lifetime severity of entrapment and anxiety symptoms at different levels of functional connectivity within the default network. The association between (C) total lifetime severity of entrapment, (D) functional connectivity between the frontoparietal and default networks and the sums of marginal fit and conditional residuals from the fitted LME models predicting depression symptoms at two 6-month follow-ups. Different colors represent different participants.

Results from the optimal unified model for anxiety revealed that total lifetime severity of entrapment at baseline was positively associated with anxiety symptoms at two follow-up assessments (*β* = 0.36, *p* = 3.10×10^−4^), and the association was stronger when RSFC within the default network was higher (Table 2; Figure 3A&C). When considering depression, the optimal unified model indicated that RSFC between the frontoparietal and default network (*β* = 0.18, *p* = 8.20*×*10^−4^) and total lifetime severity of entrapment stressor exposure (*β* = 0.17, *p* = 0.05) at baseline were positively associated with depression symptoms at two follow-up assessments (Table 3; Figure 3B&D). These results demonstrate that entrapment severity remained to be a shared predictor for both anxiety and depression even after accounting for main and interaction effects involving within- and between-network RSFC metrics, further supporting its importance in predicting both anxiety and depression.

## Discussion

Despite a wealth of prior research examining associations between stress, neurobiology and internalizing psychopathology, it remains unknown if the lifetime severity of distinct life stressor characteristics differentially predicts future anxiety and depression in adolescence, and if such prospective associations are moderated by large-scale network connectivity. To investigate, we acquired functional neuroimaging at baseline and tracked a sample of adolescents longitudinally for one year, more than half of whom were currently diagnosed with at least one depressive or anxiety disorder at time of initial assessment. We first tested our hypotheses associating lifetime severity of specific stressor characteristics differentially with anxiety or depression symptoms at two 6-month follow-up. We then explored if distinct stressor characteristics and RSFC within and between large-scale networks acted independently or jointly to predict prospective anxiety and depression symptoms.

Results from the hypothesis-driven LME models revealed that (a) higher total lifetime severity of physical danger and humiliation predicted higher levels of anxiety but not depression symptoms; (b) higher total lifetime severity of entrapment predicted higher levels of both anxiety and depression symptoms; and (c) total lifetime severity of interpersonal loss predicted neither anxiety nor depression symptoms. Results from the exploratory unified LME models additionally included fixed and interaction effects of RSFC metrics within and between large-scale functional networks and showed that (a) the main effects of higher total lifetime severity of entrapment still predicted higher levels of both anxiety and depression symptoms at the two 6-month follow-ups; (b) more positive RSFC between the frontoparietal and the default networks uniquely predicted higher levels of depression symptoms at the two 6-month follow-ups; and (c) the association between total lifetime severity of entrapment and anxiety symptoms at the two 6-month follow-ups was more pronounced (i.e., more positive) among adolescents who had more positive RSFC within the default network. These results thus support the importance of including major life stressors, as well as both RSFC within and between large-scale functional networks, in models aiming to predict changes in anxiety and depression in adolescence over time.

The results from the hypothesis-driven LME models are consistent with the prior studies suggesting that exposure to major life stressors characterized by danger, which implies threat to one’s physical integrity, is a specific risk factor for anxiety (14,16,17,19). However, these data did not reveal unique associations between loss, humiliation, and depression, as has been previously theorized (14–18). Such a discrepancy may have arisen from differences in sample characteristics. For example, although most prior studies have focused on adults recruited from the community, we used an adolescent sample, around 60% of whom were already diagnosed with anxiety and depressive disorders at the time of baseline assessment. Indeed, having formal diagnoses of anxiety and depressive disorders are strong predictors of prospective anxiety and depression symptoms (Supplemental Tables 5–12) and may have obscured the associations between loss, humiliation and depression. Nevertheless, the fixed effects of total lifetime entrapment severity were not obscured by including anxiety and depression diagnoses in the LME models predicting prospective anxiety and depression symptoms, implying that entrapment severity predicts both anxiety and depression regardless of diagnostic status.

These results are consistent with prior studies implying entrapment as a shared risk factor for both anxiety and depression (14,20). Exposure to entrapment, which refers to chronic difficulties that are unlikely to be resolved, has been associated with the onset of depressive (54) or mixed major depression-generalized anxiety episode (14). Self-reported feelings of entrapment have been associated with longitudinal changes in anxiety and depression symptoms at 1-year follow-up in clinically depressed and healthy adolescents (20). Results from the present study are thus consistent with these findings by showing that higher total lifetime severity of entrapment exposure was associated with higher levels of anxiety and depression symptoms at two 6-month follow-up assessments, highlighting entrapment as the most central shared risk factor for anxiety and depression among all stressor characteristics we examined.

In turn, results from the unified LME model predicting anxiety symptoms showed that higher total lifetime severity of entrapment exposure at baseline prospectively predicted even higher anxiety symptoms at the follow-ups for among participants who had more positive RSFC within the default network. A meta-analysis found converging evidence that hyperconnectivity within the default network was associated with anxiety symptoms across multiple anxiety disorders, including social anxiety, generalized anxiety and panic disorders (79). Findings from this meta-analysis are consistent with our results, where anxiety symptoms were computed by summing across subscales related to separation anxiety, social phobia, generalized anxiety and panic disorder symptoms. A few other studies have found that more positive intra-network RSFC within the default network in anxiety disorder (80,81). Aspects of the default network, including the posterior cingulate and the precuneus, are hypothesized to support internally-related cognition (82–84) such as rumination. Therefore, although speculative, participants displaying hyperconnectivity within the default network may exhibit higher tendency to engage in anxiety-related internal rumination and experience higher levels of anxiety symptoms over time after exposure to stressful life events (85,86).

Finally, results from the unified LME model predicting depression symptoms showed that total lifetime entrapment severity did not interact with any RSFC metric, suggesting that entrapment severity and RSFC within and between functional networks independently predicted prospective depression symptoms. The model also revealed a positive fixed effect of RSFC between the frontoparietal and the default networks in predicting prospective depression symptoms. This finding is consistent with prior studies finding hyperconnectivity between the default and the frontoparietal network in depressed adolescents and adults (46,87–89). Since anti-correlation between the default and the frontoparietal networks is theorized to reflect competition between internally-oriented and externally-oriented modes (90), the more positive RSFC between the default and the frontoparietal networks may reflect difficulty disengaging from internally-oriented thoughts to meet executive demands (91).

### Strengths and Limitations

Several strengths and limitations of this work should be noted. In terms of strengths, we leveraged a longitudinal sample of adolescents enriched for clinical diagnosis of depressive and anxiety disorders with a narrow age range. This enabled us to examine associations between exposure to major life stressors, neurobiology, and clinical symptoms during the developmental stage characterized by heightened vulnerability to stress-related psychopathology (6–10). We also assessed exposure to a variety of different types of theoretically relevant stressors across the entire life course using distinct dimensions of social-psychological characteristics. Lastly, we included all five social-psychological characteristics, all six RSFC metrics within and between the three *a priori* functional networks, as well as the potential confounding variables into the same LME model when predicting anxiety/depression symptoms at follow-up. This ensures that any prospective association between a stressor characteristic or RSFC metric and clinical symptom is robust to the inclusion of other inter-correlated stressor characteristics or RSFC metrics.

Several limitations should also be noted. First, the sample was relatively small, which limits the statistical power to detect the complex associations between stressful life event exposure, RSFC patterns, and clinical symptoms. Although this is a common issue with richly-phenotyped, longitudinal datasets involving clinical samples, findings from the present study should be validated by larger samples in future studies. Since the fMRI protocol of the current dataset was harmonized with other HCP studies (64), other HCP datasets may provide valuable resources for this purpose. Second, the vast majority (95.33%) of participants were White, and additional research is needed to examine the generalizability of these findings to other populations and demographic groups. Third, considering the limited sample size, our hypothesis-driven analyses focused on RSFC within and between the default, frontoparietal and ventral attention networks. RSFC involving other functional networks such as the dorsal attention and the limbic networks as well as the subcortical structures such as the amygdala have also been associated with anxiety and depression (46,92–95), and future high-powered analyses would benefit from a whole-brain approach. Fourth, although we included a number of covariates such as age, race and ethnicity, baseline symptoms, and diagnostic status in our LME models to ensure that our findings could not be explained by these confounders, other potential confounders such as medication use and family history of psychopathology were unavailable in this dataset and may be relevant.

### Conclusion

Through the use a longitudinal sample of adolescents, over half of whom met clinical cut-offs for at least one depressive or anxiety disorder, the present analyses demonstrate that greater total lifetime severity of entrapment exposure predicts higher levels of in anxiety and depression symptoms at two subsequent 6-month follow-up time points. Lifetime entrapment exposure prospectively predicted anxiety symptoms in participants with more positive default network connectivity. Heightened RSFC between the frontoparietal and the default network specifically predicted higher levels of depression symptoms at the two 6-month follow-ups. These results imply that among all characteristics of major life stressors that we examined using the STRAIN, entrapment may be the most important risk factor for both anxiety and depression. Our results also suggest that more positive connectivity in the default network may be a specific risk factor for anxiety when an individual is exposed to entrapment, and that more positive connectivity between the frontoparietal and default network may be a specific risk factor for depression.

## Supplementary Material

Supplement 1

## Figures and Tables

**Table 1.** Demographic characteristics for the final analytical sample (*n* = 107)

Baseline		
Demographics	*M/n*	*SD/%*
Age, mean ± SD	15.42	0.87
Sex, (% female)	66	61.68
Diagnostic group	*n*	*%*
Anxiety^a^	38	35.51
Control	41	38.32
Depression^b^	28	26.17
Race/ethnicity	*n*	*%*
African American	2	1.87
Asian	2	1.87
Hawaiian	1	0.93
White	102	95.33
Lifetime severity of stressor characteristics^c^	*M*	*SD*
Entrapment	11.21	10.30
Humiliation	7.60	7.18
Interpersonal loss	10.59	8.48
Physical danger	4.50	5.50
Role change/disruption	5.82	5.44
Clinical symptoms	*M*	*SD*
Anxiety symptoms^d^	24.36	17.86
Depression symptoms^e^	15.74	14.82
Six-month follow-up		
Clinical symptoms	*M*	*SD*
Anxiety symptoms^d^	23.81	17.71
Depression symptoms^e^	16.84	15.43
Twelve-month follow-up		
Clinical symptoms	*M*	*SD*
Anxiety symptoms^d^	21.35	15.61
Depression symptoms^e^	14.62	14.13

Note:

a Anxiety = having a current diagnosis of at least one anxiety disorder and no depressive disorder based on DSM-5.

b Depression = having a current diagnosis of at least one depressive disorder based on DSM-5.

c The total lifetime severity for each social-psychological stressor characteristic based on self-reported acute and chronic stressors from the Stress and Adversity Inventory for Adolescents (STRAIN). Entrapment: 0–26. Humiliation: 0–35. Interpersonal loss: 0–50. Physical danger: 0–65. Role change/disruption: 0–70

d Anxiety symptoms was computed by summing the four anxiety subscales (Separation Anxiety Disorder, Social Phobia, Generalized Anxiety and Panic Disorder) from the Revised Children’s Anxiety and Depression Scale (RCADS), ranging from 0 to 93.

e Depression symptoms was computed by the total Mood and Feelings Questionnaire (MFQ) score ranging from 0 to 66.

**Table 2.** Results of unified LME models testing if anxiety symptoms at two 6-month follow-ups can be predicted from total lifetime severity of physical danger, total lifetime severity of entrapment, and RSFC within and between functional networks at baseline

Predictor	Estimate	*SE*	95% CI		*t*	*p*
			*LL*	*UL*		
wFPN FC	−0.057	0.065	−0.17	0.053	−0.87	0.39
wVAN FC	0.076	0.063	−0.029	0.18	1.22	0.23
wDN FC	−0.024	0.060	−0.13	0.082	−0.40	0.69
bFPN-DN FC	−0.12	0.070	−0.24	−0.0014	−1.71	0.091
bFPN-VAN FC	0.089	0.059	−0.0096	0.19	1.52	0.13
bDN-VAN FC	−0.023	0.053	−0.11	0.066	−0.43	0.67
Physical danger	0.083	0.086	−0.062	0.23	0.97	0.33
**Entrapment**	**0.36**	**0.095**	**0.20**	**0.52**	**3.78**	**0.0031** ***
**Baseline depression symptoms**	**0.41**	**0.093**	**0.25**	**0.57**	**4.42**	**3.14*×*10^−5^** ****
Diagnosis of Depression	−0.032	0.18	−0.34	0.28	−0.17	0.86
**Diagnosis of Anxiety**	**0.46**	**0.13**	**0.24**	**0.67**	**3.59**	**0.00057** ****
White	0.016	0.51	−0.84	0.87	0.032	0.97
African American	0.19	0.62	−0.86	1.24	0.30	0.76
Asian	−0.060	0.62	−1.10	0.98	−0.097	0.92
Ethnic group	−0.28	0.20	−0.62	0.065	−1.36	0.18
Baseline age	0.072	0.054	−0.021	0.17	1.34	0.18
**Sex**	−**0.30**	**0.11**	−**0.49**	−**0.11**	−**2.70**	**0.0085** **
**Time**	−**0.088**	**0.037**	−**0.16**	−**0.015**	−**2.37**	**0.020** *
wFPN FC:Physical danger	0.17	0.094	0.0076	0.33	1.77	0.080
wFPN FC:Entrapment	−0.15	0.086	−0.30	−0.010	−1.80	0.076
wVAN FC:Physical danger	−0.029	0.069	−0.15	0.088	−0.42	0.68
wVAN FC:Entrapment	0.012	0.076	−0.12	0.14	0.15	0.88
wDN FC:Physical danger	−0.10	0.064	−0.22	0.0074	−1.63	0.11
**wDN FC:Entrapment**	**0.27**	**0.077**	**0.14**	**0.40**	**3.52**	**0.00073** ****
bFPN-DN FC:Physical danger	−0.0039	0.12	−0.21	0.20	−0.033	0.97
bFPN-DN FC:Entrapment	−0.022	0.11	−0.21	0.16	−0.21	0.84
bFPN-VAN FC:Physical danger	−0.14	0.088	−0.29	0.0026	−1.65	0.10
bFPN-VAN FC:Entrapment	0.10	0.094	−0.058	0.26	1.07	0.29
bDN-VAN FC:Physical danger	0.045	0.086	−0.10	0.19	0.52	0.60
bDN-VAN FC:Entrapment	0.048	0.066	−0.063	0.16	0.73	0.47
Intercept	−0.024	0.51	−0.88	0.83	−0.047	0.96

Note.

* *p* < 0.05;

** *p* < 0.01;

*** *p* < 0.005;

**** *p* < 0.001.

FC = functional connectivity. bFPN-DN = between frontoparietal and default network, bDN-VAN = between default network and ventral attention network. bFPN-VAN = between frontoparietal and ventral attention network. wFPN = within frontoparietal network. wDN = within default network. wVAN = within ventral attention network

**Table 3.** Results of the unified LME model testing if depression symptoms at two 6-month follow-ups can be predicted from total lifetime severity of entrapment and RSFC within and between functional networks at baseline

Predictor	Estimate	*SE*	95% CI		*t*	*p*
			*LL*	*UL*		
wFPN FC	0.089	0.065	−0.025	0.20	1.37	0.17
wVAN FC	−0.086	0.061	−0.19	0.021	−1.41	0.16
wDN FC	0.088	0.059	−0.015	0.19	1.50	0.14
**bFPN-DN FC**	**0.18**	**0.067**	**0.064**	**0.30**	**2.71**	**0.0082** **
bFPN-VAN FC	−0.049	0.056	−0.15	0.050	−0.87	0.39
bDN-VAN FC	0.0066	0.053	−0.087	0.10	0.12	0.90
**Entrapment**	**0.17**	**0.086**	**0.023**	**0.32**	**2.02**	**0.046** *
**Baseline anxiety symptoms**	**0.45**	**0.091**	**0.29**	**0.61**	**4.98**	**3.35×10^−6^** ****
**Diagnose of Depression**	**0.58**	**0.16**	**0.31**	**0.86**	**3.71**	**0.000372** ****
Diagnosis of Anxiety	−0.028	0.14	−0.27	0.22	−0.20	0.84
White	−0.55	0.51	−1.45	0.34	−1.08	0.28
African American	0.50	0.64	−0.62	1.61	0.78	0.44
Asian	−0.60	0.63	−1.71	0.52	−0.94	0.35
Ethnic group	0.25	0.21	−0.11	0.62	1.22	0.22
**Baseline age**	**0.12**	**0.053**	**0.031**	**0.22**	**2.34**	**0.022** *
Sex	−0.048	0.11	−0.25	0.15	−0.41	0.68
Time	−0.038	0.034	−0.11	0.029	−1.11	0.27
wFPN FC:Entrapment	−0.070	0.068	−0.19	0.048	−1.04	0.30
wVAN FC:Entrapment	−0.025	0.065	−0.14	0.089	−0.39	0.70
wDN FC:Entrapment	0.015	0.061	−0.092	0.12	0.25	0.80
bFPN-DN FC:Entrapment	0.056	0.086	−0.096	0.21	0.65	0.52
bFPN-VAN FC:Entrapment	−0.068	0.056	−0.17	0.031	−1.21	0.23
bDN-VAN FC:Entrapment	−0.0026	0.055	−0.098	0.093	−0.047	0.96
Intercept	0.39	0.51	−0.51	1.29	0.76	0.45

Note.

* *p* < 0.05;

** *p* < 0.01;

*** *p* < 0.005;

**** *p* < 0.001.

FC = functional connectivity. bFPN-DN = between frontoparietal and default network, bDN-VAN = between default network and ventral attention network. bFPN-VAN = between frontoparietal and ventral attention network. wFPN = within frontoparietal network. wDN = within default network. wVAN = within ventral attention network
