# Supplementary material for: Shared and unique lifetime stressor characteristics and network connectivity predict adolescent anxiety and depression
Source: bioRxiv. 2024 Dec 1:2024.10.25.620373. Originally published 2024 Oct 26. Preprint. [Version 2] doi: 10.1101/2024.10.25.620373 (PMC11527110; doi:10.1101/2024.10.25.620373)
Supplement: Supplement 1 [file media-1.docx]

Supplementary Materials for

**Shared and unique lifetime stressor characteristics and network connectivity predict adolescent anxiety and depression**

Yueyue Lydia Qu^1,2^, Sidhant Chopra^3,4^, Shijie Qu^1,2^, Carrisa V. Cocuzza^5^, Loïc Labache^5^, Clemens C.C. Bauer ^6,7,8^, Francesca Morfini^6,7^, Susan Whitfield-Gabrieli^6,7,8^, George M. Slavich^9^, Jutta Joormann^1,2^, & Avram J. Holmes^5^

^1^ Department of Psychology, Yale University, New Haven, CT, USA

^2^ Wu Tsai Institute, Yale University, New Haven, CT, USA

^3^ Orygen, Melbourne, VIC, Australia

^4^ Centre for Youth Mental Health, The University of Melbourne, Melbourne, VIC, Australia

^5^ Department of Psychiatry, Brain Health Institute, Rutgers University, Piscataway, NJ, USA

^6^ Department of Psychology, Northeastern University, Boston, MA, USA

^7^ Center for Cognitive & Brain Health, Northeastern University, Boston, MA, USA

^8^ Department of Brain and Cognitive Sciences and McGovern Institute for Brain Research, Massachusetts Institute of Technology, Cambridge, MA, USA

^9^ Department of Psychiatry and Biobehavioral Sciences, University of California, Los Angeles, CA, USA

**Supplemental Result 1. Differences in symptom levels across diagnostic groups**

Overall, participants across the three diagnostic groups exhibited different levels of anxiety and depression symptoms across all three time points (**Supplementary Figure 1**). One-way ANOVA revealed a significant effect of diagnostic group on the level of depression symptoms across all three time points (Baseline: *F*(2,104) = 55.66, *p* < 10^-16^; 6-month follow-up: *F* (2,104) = 26.02, *p* = 6.86×10^-10^; 12-month follow-up: *F*(2,104) = 25.49, *p* = 9.83×10^-10^). Post-hoc comparisons using the Tukey’s test (**Supplemental Table 2**) revealed that the mean level of depression symptoms for the Depression group (Baseline: 31.89 (Mean) ± 13.93 (SD); 6-month follow-up: 29.86 ± 14.18; 12-month follow-up: 25.93 ± 14.91) was higher than the Anxiety group (Baseline: 15.42 ±11.55; 6-month follow-up: 17.45±14.02; 12-month follow-up: 15.97±13.40) which was also higher than the Control group (Baseline: 5.00 ± 5.17; 6-month follow-up: 7.39 ± 10.16; 12-month follow-up: 5.63 ± 6.24). A similar pattern was observed for the level of anxiety symptoms. One-way ANOVA revealed an effect of diagnostic group on the level of anxiety symptoms across all three time points (Baseline: *F*(2,104) = 36.68, *p* = 8.84×10^-13^; 6-month follow-up: *F*(2,104) = 22.99, *p* = 5.39×10^-9^; 12-month follow-up: *F*(2,104) = 21.58, *p* = 1.45×10^-8^). Post-hoc Tukey’s test (**Supplemental Table 2**) showed that the mean level of anxiety symptoms for the Depression group (Baseline: 37.50 ± 18.09; 6-month follow-up: 35.50 ± 18.41; 12-month follow-up: 31.04±14.92) was not different from the Anxiety group (Baseline: 29.79 ± 15.17; 6-month follow-up: 27.95 ± 16.02; 12-month follow-up: 25.32 ± 15.02). Both groups exhibited higher levels of anxiety symptoms than the Control group (Baseline: 10.37 ± 7.86; 6-month follow-up: 12.00 ± 10.50; 12-month follow-up: 11.05 ± 9.88).

**Supplementary Table 1**. Bivariate correlations between continuous variables in the LME models

|  | **Anxiety at time 1^a^** | **Anxiety at time 2^b^** | **Depression at baseline** | **Depression at time 1 ^a^** | **Depression at time 2 ^b^** | **Interpersonal loss** | **Physical danger** | **Entrapment** | **Role reversal** | **Humiliation** | **wFPN FC** | **wDN FC** | **wVAN FC** | **bFPN-DN FC** | **bFPN-VAN FC** | **bDN-VAN FC** | **Baseline Age** |
| --- | --- | --- | --- | --- | --- | --- | --- | --- | --- | --- | --- | --- | --- | --- | --- | --- | --- |
| **Anxiety at baseline** | **0.81****** | **0.7****** | **0.79****** | **0.71****** | **0.67****** | **0.48****** | **0.63****** | **0.77****** | **0.62****** | **0.7****** | 0 | -0.01 | 0.11 | 0.15 | 0 | -0.04 | 0.17 |
| **Anxiety at time 1^a^** |  | **0.84****** | **0.62****** | **0.82****** | **0.71****** | **0.34****** | **0.45****** | **0.67****** | **0.57****** | **0.57****** | 0.07 | 0.02 | 0.11 | 0.09 | -0.04 | -0.12 | **0.3***** |
| **Anxiety at time 2^b^** |  |  | **0.59****** | **0.71****** | **0.84****** | **0.27***** | **0.41****** | **0.59****** | **0.49****** | **0.45****** | 0.05 | 0.08 | 0.13 | 0.17 | -0.05 | -0.11 | **0.28***** |
| **Depression at baseline** |  |  |  | **0.75****** | **0.73****** | **0.49****** | **0.49****** | **0.66****** | **0.49****** | **0.59****** | -0.06 | -0.03 | 0.07 | **0.31****** | 0 | 0 | 0.2 |
| **Depression at time 1^a^** |  |  |  |  | **0.78****** | **0.44****** | **0.5****** | **0.65****** | **0.5****** | **0.55****** | 0.06 | 0.01 | 0.09 | **0.25**** | -0.04 | -0.09 | **0.41****** |
| **Depression at time 2^b^** |  |  |  |  |  | **0.33****** | **0.41****** | **0.6****** | **0.42****** | **0.46****** | 0.05 | 0.08 | 0.05 | **0.21*** | -0.06 | 0.02 | **0.29***** |
| **Interpersonal loss** |  |  |  |  |  |  | **0.41****** | **0.58****** | **0.55****** | **0.51****** | 0.09 | 0.03 | 0.12 | 0.1 | 0.02 | -0.14 | **0.22***** |
| **Physical danger** |  |  |  |  |  |  |  | **0.59****** | **0.56****** | **0.68****** | 0.09 | 0.15 | 0.22* | 0.09 | 0 | -0.13 | 0.09 |
| **Entrapment** |  |  |  |  |  |  |  |  | **0.77****** | **0.79****** | 0.17 | 0.14 | 0.05 | 0.02 | -0.06 | -0.12 | **0.23***** |
| **Role reversal** |  |  |  |  |  |  |  |  |  | **0.64****** | 0.17 | 0.15 | 0.12 | 0.04 | 0 | -0.17 | **0.25***** |
| **Humiliation** |  |  |  |  |  |  |  |  |  |  | 0.02 | 0.05 | 0.18 | 0.1 | -0.04 | -0.18 | 0.1 |
| **wFPN FC** |  |  |  |  |  |  |  |  |  |  |  | **0.42****** | 0.09 | **-0.53****** | **-0.29***** | 0.07 | -0.03 |
| **wDN FC** |  |  |  |  |  |  |  |  |  |  |  |  | 0.17 | **-0.34****** | -0.12 | -0.13 | -0.11 |
| **wVAN FC** |  |  |  |  |  |  |  |  |  |  |  |  |  | 0.2* | **-0.37****** | **-0.23*** | -0.09 |
| **bFPN-DN FC** |  |  |  |  |  |  |  |  |  |  |  |  |  |  | -0.06 | 0 | **0.26**** |
| **bFPN-VAN FC** |  |  |  |  |  |  |  |  |  |  |  |  |  |  |  | -0.02 | -0.02 |
| **bDN-VAN FC** |  |  |  |  |  |  |  |  |  |  |  |  |  |  |  |  | 0.02 |

Note: All statistics are Pearson's correlation coefficient r. **p*<.05 ***p*<0.01****p*<0.005 *****p*<0.001 a. Time 1 = at 6-month follow-up assessment. b. Time 2 = at 12-month follow-up assessment. FC = functional connectivity. bFPN-DN = between frontoparietal and default network, bDN-VAN = between default network and ventral attention network. bFPN-VAN = between frontoparietal and ventral attention network. wFPN = within frontoparietal network. wDN = within default network. wVAN = within ventral attention network

**Supplemental Table 2.** Results for Tukey’s test for comparing depressive and anxiety symptoms between every pair of diagnostic groups at baseline, 6-month follow-up assessment and 12-month follow-up assessment. Anxiety = having a current diagnosis of at least one anxiety disorder and no depressive disorder; Depression=having a current diagnosis of at least one depressive disorder; Control=having no current or lifetime diagnosis of any psychiatric disorder.

| Baseline | | | | | |
| --- | --- | --- | --- | --- | --- |
| Depression symptom | | | Anxiety symptom | | |
| Comparison | Difference | *p* | Comparison | Difference | *p* |
| Depression - Control | 26.89 | **<10^-16^** | Depression - Control | 27.13 | **<10^-16^** |
| Anxiety - Control | 10.42 | **6.38×10^-5^** | Anxiety - Control | 19.42 | **<10^-16^** |
| Depression - Anxiety | 16.47 | **<10^-16^** | Depression - Anxiety | 7.71 | 0.069 |
| Six-month follow-up | | | | | |
| Depression symptom | | | Anxiety symptom | | |
| Comparison | Difference | *p* | Comparison | Difference | *p* |
| Depression - Control | 22.47 | **<10^-16^** | Depression - Control | 23.50 | **<10^-16^** |
| Anxiety - Control | 10.06 | **0.0019** | Anxiety - Control | 15.95 | **0.000019** |
| Depression - Anxiety | 12.41 | **0.00047** | Depression - Anxiety | 7.55 | 0.11 |
| Twelve-month follow-up | | | | | |
| Depression symptom | | | Anxiety symptom | | |
| Comparison | Difference | *p* | Comparison | Difference | *p* |
| Depression - Control | 20.29 | **<10^-16^** | Depression - Control | 19.99 | **<10^-16^** |
| Anxiety - Control | 10.34 | **0.00045** | Anxiety - Control | 14.27 | **0.000017** |
| Depression - Anxiety | 9.95 | **0.0026** | Depression - Anxiety | 5.72 | 0.20 |

**Supplemental Table 3.** Conditional AIC for hypothesis-driven LME models testing associations between prospective level of anxiety/depression symptom and total lifetime severity of each stressor social-psychological characteristic at baseline

| Symptom ~ stressor characteristic | Conditional AIC (random intercept) | Conditional AIC (random slope + intercept) |
| --- | --- | --- |
| Anxiety ~ Physical danger | 515.40 | **446.60** |
| Depression ~ Physical danger | **545.19** | 545.19  (singular model) |
| Anxiety ~ Interpersonal loss | 518.24 | **451.26** |
| Depression ~ Interpersonal loss | **544.81** | 544.81  (singular model) |
| Anxiety ~ Humiliation | 514.70 | **444.74** |
| Depression ~ Humiliation | **545.11** | 545.11  (singular model) |
| Anxiety ~ Entrapment | 512.14 | **444.05** |
| Depression ~ Entrapment | **543.79** | 543.79  (singular model) |

**Supplemental Table 4.** Results of the hypothesis-driven LME model testing if anxiety symptom at two 6-month follow-ups can be predicted from total lifetime severity of physical danger at baseline

| Predictor | Estimate | | *SE* | 95% CI | | *t* | *p* |
| --- | --- | --- | --- | --- | --- | --- | --- |
|  | |  |  | *LL* | *UL* |  |  |
| **Physical danger** | **0.25** | | **0.058** | **0.14** | **0.36** | **4.33** | **3.63×10^-5^****** |
| **Baseline depression symptoms** | **0.45** | | **0.080** | **0.29** | **0.61** | **5.64** | **1.74×10^-^****^7^****** |
| DepressionDiagnosis | 0.27 | | 0.19 | -0.094 | 0.62 | 1.39 | 0.17 |
| **AnxietyDiagnosis** | **0.59** | | **0.13** | **0.35** | **0.84** | **4.51** | **1.85×10^-5^****** |
| White | -0.40 | | 0.53 | -1.39 | 0.60 | -0.75 | 0.46 |
| African American | -0.069 | | 0.65 | -1.29 | 1.15 | -0.11 | 0.92 |
| Asian | -0.67 | | 0.65 | -1.88 | 0.55 | -1.02 | 0.31 |
| Ethnic group | -0.22 | | 0.21 | -0.61 | 0.17 | 1.07 | 0.29 |
| Baseline age | 0.0098 | | 0.054 | -0.094 | 0.11 | 0.18 | 0.86 |
| **Sex** | **-0.41** | | **0.12** | **-0.62** | **-0.19** | **-3.53** | **0.00063****** |
| **Time** | **-0.088** | | **0.037** | **-0.16** | **-0.015** | **-2.37** | **0.020*** |
| Intercept | 0.28 | | 0.54 | -0.72 | 1.29 | 0.53 | 0.60 |

Note. **p* < 0.05; ***p* < 0.01; ****p* < 0.005; *****p* < 0.001. The LME model was fitted with a random slope plus a random intercept.

**Supplemental Table 5.** Results of the hypothesis-driven LME model testing if depression symptom at two 6-month follow-ups can be predicted from total lifetime severity of physical danger at baseline

| Predictor | Estimate | *SE* | 95% CI | | *t* | *p* |
| --- | --- | --- | --- | --- | --- | --- |
|  |  |  | *LL* | *UL* |  |  |
| Physical danger | -0.0079 | 0.062 | -0.12 | 0.11 | -0.13 | 0.90 |
| **Baseline anxiety symptoms** | **0.56** | **0.079** | **0.41** | **0.71** | **7.08** | **2.43×10^-10^****** |
| **DepressionDiagnosis** | **0.65** | **0.16** | **0.36** | **0.94** | **4.16** | **6.95×10^-5^****** |
| AnxietyDiagnosis | -0.020 | 0.14 | -0.27 | 0.23 | -0.15 | 0.88 |
| White | -0.13 | 0.49 | -1.05 | 0.79 | -0.27 | 0.79 |
| African American | 0.61 | 0.60 | -0.52 | 1.73 | 1.01 | 0.32 |
| Asian | 0.15 | 0.60 | -0.98 | 1.28 | 0.24 | 0.81 |
| Ethnic group | 0.23 | 0.19 | -0.13 | 0.59 | 1.22 | 0.23 |
| **Baseline age** | **0.16** | **0.050** | **0.066** | **0.25** | **3.18** | **0.0020***** |
| Sex | -0.070 | 0.11 | -0.28 | 0.14 | -0.63 | 0.53 |
| Time | -0.038 | 0.034 | -0.11 | 0.029 | -0.63 | 0.53 |
| Intercept | -0.04 | 0.50 | -0.97 | 0.89 | -0.082 | 0.93 |

Note. **p* < 0.05; ***p* < 0.01; ****p* < 0.005; *****p* < 0.001. The LME model was fitted with a random intercept.

**Supplemental Table 6.** Results of the hypothesis-driven LME model testing if anxiety symptom at two 6-month follow-ups can be predicted from total lifetime severity of interpersonal loss at baseline

| Predictor | Estimate | *SE* | 95% CI | | *t* | *p* |
| --- | --- | --- | --- | --- | --- | --- |
|  |  |  | *LL* | *UL* |  |  |
| Interpersonal loss | 0.039 | 0.064 | -0.086 | 0.16 | 0.60 | 0.55 |
| **Baseline depression symptoms** | **0.50** | **0.091** | **0.32** | **0.68** | **5.48** | **3.48×10^-7^****** |
| DepressionDiagnosis | 0.38 | 0.21 | -0.015 | 0.77 | 1.80 | 0.075 |
| **AnxietyDiagnosis** | **0.62** | **0.14** | **0.35** | **0.89** | **4.33** | **3.62×10^-5^****** |
| White | -0.22 | 0.58 | -1.30 | 0.87 | -0.38 | 0.71 |
| African American | 0.066 | 0.70 | -1.26 | 1.39 | 0.094 | 0.93 |
| Asian | -0.36 | 0.70 | -1.67 | 0.96 | -0.51 | 0.61 |
| Ethnic group | -0.24 | 0.23 | -0.66 | 0.19 | -1.04 | 0.30 |
| Baseline age | 0.0067 | 0.059 | -0.11 | 0.12 | 0.11 | 0.91 |
| **Sex** | **-0.44** | **0.12** | **-0.68** | **-0.21** | **-3.57** | **0.00057****** |
| **Time** | **-0.088** | **0.037** | **-0.16** | **-0.015** | **-2.37** | **0.020*** |
| Intercept | 0.079 | 0.58 | -1.02 | 1.18 | 0.14 | 0.89 |

Note. **p* < 0.05; ***p* < 0.01; ****p* < 0.005; *****p* < 0.001. The LME model was fitted with a random slope plus a random intercept.

**Supplemental Table 7.** Results of the hypothesis-driven LME model testing if depression symptom at two 6-month follow-ups can be predicted from total lifetime severity of interpersonal loss at baseline

| Predictor | Estimate | *SE* | 95% CI | | *t* | *p* |
| --- | --- | --- | --- | --- | --- | --- |
|  |  |  | *LL* | *UL* |  |  |
| Interpersonal loss | 0.057 | 0.054 | -0.044 | 0.16 | 1.05 | 0.29 |
| **Baseline anxiety symptoms** | **0.53** | **0.071** | **0.39** | **0.66** | **7.36** | **6.13×10^-11^****** |
| **DepressionDiagnosis** | **0.66** | **0.14** | **0.37** | **0.95** | **4.23** | **5.31×10^-5^****** |
| AnxietyDiagnosis | -0.053 | 0.13 | -0.25 | 0.24 | -0.040 | 0.97 |
| White | -0.097 | 0.50 | -1.01 | 0.82 | -0.20 | 0.84 |
| African American | 0.61 | 0.60 | -0.51 | 1.72 | 1.02 | 0.31 |
| Asian | 0.17 | 0.59 | -0.94 | 1.28 | 0.29 | 0.78 |
| Ethnic group | 0.23 | 0.19 | -0.13 | 0.59 | 1.20 | 0.23 |
| **Baseline age** | **0.15** | **0.051** | **0.055** | **0.25** | **2.96** | **0.0039***** |
| Sex | -0.070 | 0.11 | -0.28 | 0.13 | -0.63 | 0.53 |
| Time | -0.038 | 0.034 | -0.11 | 0.029 | -1.11 | 0.27 |
| Intercept | -0.078 | 0.40 | -1.00 | 0.85 | -0.16 | 0.87 |

Note. **p* < 0.05; ***p* < 0.01; ****p* < 0.005; *****p* < 0.001. The LME model was fitted with a random intercept.

**Supplemental Table 8.** Results of the hypothesis-driven LME model testing if anxiety symptom at two 6-month follow-ups can be predicted from total lifetime severity of humiliation at baseline

| Predictor | Estimate | *SE* | *95% CI* | | *t* | *p* |
| --- | --- | --- | --- | --- | --- | --- |
|  |  |  | *LL* | *UL* |  |  |
| **Humiliation** | **0.31** | **0.063** | **0.19** | **0.44** | **5.00** | **2.56×10^-6^****** |
| **Baseline depression symptoms** | **0.39** | **0.082** | **0.23** | **0.55** | **4.69** | **9.00×10^-6^****** |
| DepressionDiagnosis | 0.28 | 0.21 | -0.067 | 0.63 | 1.52 | 0.13 |
| **AnxietyDiagnosis** | **0.54** | **0.13** | **0.30** | **0.78** | **4.16** | **6.96×10^-5^****** |
| White | -0.49 | 0.52 | -1.47 | 0.48 | -0.94 | 0.35 |
| African American | -0.31 | 0.64 | -1.51 | 0.89 | -0.49 | 0.63 |
| Asian | -0.91 | 0.64 | -2.11 | 0.29 | -1.42 | 0.16 |
| Ethnic group | -0.36 | 0.20 | -0.74 | 0.026 | -1.75 | 0.083 |
| Baseline age | 0.011 | 0.053 | -0.090 | 0.11 | 0.20 | 0.84 |
| **Sex** | **-0.41** | **0.11** | **-0.62** | **-0.20** | **-3.67** | **0.00039****** |
| **Time** | **-0.088** | **0.037** | **-0.16** | **-0.015** | **-2.37** | **0.020*** |
| Intercept | 0.41 | 0.53 | -0.58 | 1.39 | 0.77 | 0.44 |

Note. **p* < 0.05; ***p* < 0.01; ****p* < 0.005; *****p* < 0.001. The LME model was fitted with a random slope plus a random intercept.

**Supplemental Table 9.** Results of the hypothesis-driven LME model testing if depression symptom at two 6-month follow-ups can be predicted from total lifetime severity of humiliation at baseline

| Predictor | Estimate | *SE* | 95% CI | | *t* | *p* |
| --- | --- | --- | --- | --- | --- | --- |
|  |  |  | *LL* | *UL* |  |  |
| Humiliation | 0.034 | 0.068 | -0.093 | 0.16 | 0.50 | 0.62 |
| **Baseline anxiety symptoms** | **0.53** | **0.083** | **0.37** | **0.69** | **6.35** | **7.25×10^-9^****** |
| **DepressionDiagnosis** | **0.64** | **0.16** | **0.35** | **0.94** | **4.15** | **7.30×10^-5^****** |
| AnxietyDiagnosis | -0.011 | 0.13 | -0.26 | 0.24 | -0.083 | 0.93 |
| White | -0.17 | 0.49 | -1.10 | 0.75 | -0.35 | 0.73 |
| African American | 0.56 | 0.60 | -0.57 | 1.69 | 0.92 | 0.36 |
| Asian | 0.070 | 0.61 | -1.07 | 1.21 | 0.12 | 0.91 |
| Ethnic group | 0.22 | 0.20 | -0.15 | 0.58 | 1.10 | 0.27 |
| **Baseline age** | **0.16** | **0.050** | **0.066** | **0.25** | **3.18** | **0.0020***** |
| Sex | -0.070 | 0.11 | -0.29 | 0.13 | -0.69 | 0.49 |
| Time | -0.038 | 0.034 | -0.11 | 0.029 | -1.11 | 0.27 |
| Intercept | 0.0021 | 0.50 | -0.93 | 0.94 | 0.004 | 1.00 |

Note. **p* < 0.05; ***p* < 0.01; ****p* < 0.005; *****p* < 0.001. The LME model was fitted with a random intercept.

**Supplemental Table 10.** Results of the hypothesis-driven LME model testing if anxiety symptom at two 6-month follow-ups can be predicted from total lifetime severity of entrapment at baseline

| Predictor | Estimate | *SE* | *95% CI* | | *t* | *p* |
| --- | --- | --- | --- | --- | --- | --- |
|  |  |  | *LL* | *UL* |  |  |
| **Entrapment** | **0.39** | **0.065** | **0.26** | **0.51** | **5.91** | **5.20×10^-8^****** |
| **Baseline depression symptoms** | **0.31** | **0.082** | **0.16** | **0.47** | **3.86** | **0.00021****** |
| DepressionDiagnosis | 0.33 | 0.18 | -0.0061 | 0.66 | 1.85 | 0.068 |
| **AnxietyDiagnosis** | **0.48** | **0.13** | **0.25** | **0.72** | **3.86** | **0.00021****** |
| White | -0.21 | 0.49 | -1.14 | 0.72 | -0.43 | 0.67 |
| African American | 0.28 | 0.61 | -0.86 | 1.42 | 0.46 | 0.65 |
| Asian | -0.54 | 0.60 | -1.67 | 0.60 | -0.89 | 0.38 |
| Ethnic group | -0.19 | 0.19 | -0.55 | 0.18 | -0.96 | 0.34 |
| Baseline age | -0.016 | 0.051 | -0.11 | 0.081 | -0.32 | 0.75 |
| **Sex** | **-0.35** | **0.11** | **-0.56** | **-0.15** | **-3.24** | **0.0016***** |
| **Time** | **-0.088** | **0.037** | **-0.16** | **-0.015** | **-2.37** | **0.020*** |
| Intercept | 0.096 | 0.50 | -0.84 | 1.04 | 0.19 | 0.85 |

Note. **p* < 0.05; ***p* < 0.01; ****p* < 0.005; *****p* < 0.001. The LME model was fitted with a random slope plus a random intercept.

**Supplemental Table 11.** Results of the hypothesis-driven LME model testing if depression symptom at two 6-month follow-ups can be predicted from total lifetime severity of entrapment at baseline

| Predictor | Estimate | *SE* | 95% CI | | *t* | *p* |
| --- | --- | --- | --- | --- | --- | --- |
|  |  |  | *LL* | *UL* |  |  |
| **Entrapment** | **0.15** | **0.074** | **0.010** | **0.29** | **2.01** | **0.047*** |
| **Baseline anxiety symptoms** | **0.44** | **0.086** | **0.28** | **0.60** | **5.13** | **1.52×10^-6^****** |
| **DepressionDiagnosis** | **0.65** | **0.15** | **0.36** | **0.93** | **4.24** | **5.10×10^-5^****** |
| AnxietyDiagnosis | -0.0060 | 0.13 | -0.25 | 0.24 | -0.046 | 0.96 |
| White | -0.15 | 0.48 | -1.04 | 0.75 | -0.31 | 0.76 |
| African American | 0.66 | 0.59 | -0.44 | 1.76 | 1.12 | 0.26 |
| Asian | 0.043 | 0.59 | -1.06 | 1.14 | 0.073 | 0.94 |
| Ethnic group | 0.23 | 0.19 | -0.12 | 0.58 | 1.22 | 0.22 |
| **Baseline age** | **0.14** | **0.050** | **0.051** | **0.24** | **2.90** | **0.0047***** |
| Sex | -0.074 | 0.11 | -0.28 | 0.13 | -0.68 | 0.50 |
| Time | -0.038 | 0.034 | -0.11 | 0.029 | -1.11 | 0.27 |
| Intercept | -0.027 | 0.48 | -0.94 | 0.88 | -0.056 | 0.96 |

Note. **p* < 0.05; ***p* < 0.01; ****p* < 0.005; *****p* < 0.001. The LME model was fitted with a random intercept.

**Supplemental Table 12**. Conditional AIC for unified LME models exploring associations between prospective levels of anxiety symptoms, different subsets of stressor characteristics and RSFC within and between functional networks

| Stressor characteristics entered into the LME model | Conditional AIC (random intercept) | Conditional AIC (random slope + intercept) | Any stress characteristic fixed effect term having VIF ≥ 5? |
| --- | --- | --- | --- |
| Physical danger + interpersonal loss + humiliation + entrapment + role change/disruption | 523.64 | 450.59 | All except interpersonal loss |
| Interpersonal loss + humiliation + entrapment + role change/disruption | 522.57 | 450.21 | All except interpersonal loss |
| Physical danger + humiliation + entrapment + role change/disruption | 520.94 | 448.40 | All |
| Physical danger + interpersonal loss + entrapment + role change/disruption | 520.28 | 448.70 | All except interpersonal loss |
| Physical danger + interpersonal loss + humiliation + role change/disruption | 521.19 | 450.71 | All except interpersonal loss |
| Physical danger + interpersonal loss + humiliation + entrapment | 520.75 | 447.34 | All except interpersonal loss |
| Physical danger + interpersonal loss + humiliation | 521.50 | 448.31 | Humiliation |
| Physical danger + interpersonal loss + role change/disruption | 521.19 | 450.71 | Physical danger |
| Physical danger + interpersonal loss + entrapment | 517.32 | 446.58 | Entrapment |
| Physical danger + humiliation + entrapment | 518.15 | 445.08 | Humiliation,  entrapment |
| Physical danger + humiliation + role change/disruption | 521.15 | 448.50 | Physical danger |
| Physical danger + entrapment + role change/disruption | 517.50 | 445.48 | All |
| Interpersonal loss + humiliation + entrapment | 520.61 | 449.23 | Entrapment, humiliation |
| Interpersonal loss + humiliation + role change/disruption | 521.38 | 448.55 | None |
| Interpersonal loss + entrapment + role change/disruption | 519.12 | 448.26 | Role change/disruption, entrapment |
| Humiliation + entrapment + role change/disruption | 519.72 | 447.62 | Humiliation, entrapment |
| **Physical danger + entrapment** | 514.63 | **443.43** | None |
| Humiliation + entrapment | 517.69 | 446.19 | Entrapment |
| Physical danger + humiliation | 518.87 | 446.11 | None |
| Physical danger | 518.27 | 448.12 | None |
| Entrapment | 514.48 | 444.96 | None |

Note: All continuous predictors and covariates were standardized before running the LME models to minimize multicollinearity. Bolded=LME model with the lowest conditional AIC

**Supplemental Table 13**. Conditional AIC for unified LME models exploring associations between prospective levels of depression symptoms, different subsets of stressor characteristics and RSFC within and between functional networks

| Stressor characteristics entered into the LME model | Conditional AIC (random intercept) | Conditional AIC (random slope + intercept) | Any stress characteristic fixed effect term having VIF ≥ 5? |
| --- | --- | --- | --- |
| Physical danger + interpersonal loss + humiliation + entrapment + role change/disruption | 560.41 | 560.41  (singular model) | All except interpersonal loss |
| Interpersonal loss + humiliation + entrapment + role change/disruption | 557.40 | 557.40  (singular model) | All except interpersonal loss |
| Physical danger + humiliation + entrapment + role change/disruption | 558.07 | 558.07  (singular model) | All |
| Physical danger + interpersonal loss + entrapment + role change/disruption | 558.58 | 558.58  (singular model) | All except interpersonal loss |
| Physical danger + interpersonal loss + humiliation + role change/disruption | 558.37 | 558.37  (singular model) | All except interpersonal loss |
| Physical danger + interpersonal loss + humiliation + entrapment | 556.99 | 540.87  (singular model) | All except interpersonal loss |
| Physical danger + interpersonal loss + humiliation | 555.59 | 555.59  (singular model) | Humiliation |
| Physical danger + interpersonal loss + role change/disruption | 555.80 | 541.96  (singular model) | Physical danger role change/disruption |
| Physical danger + interpersonal loss + entrapment | 554.66 | 554.66  (singular model) | Entrapment |
| Physical danger + humiliation + entrapment | 554.31 | 554.31  (singular model) | Humiliation, entrapment |
| Physical danger + humiliation + role change/disruption | 554.95 | 554.95  (singular model) | Physical danger, role change/disruption |
| Physical danger + entrapment + role change/disruption | 555.72 | 544.63  (singular model) | All |
| Interpersonal loss + humiliation + entrapment | 554.40 | 554.40  (singular model) | Entrapment,  humiliation |
| Interpersonal loss + humiliation + role change/disruption | 554.73 | 554.73  (singular model) | Humiliation,  role change/disruption |
| Interpersonal loss + entrapment + role change/disruption | 554.58 | 554.58  (singular model) | Role change/disruption,  entrapment |
| Humiliation + entrapment + role change/disruption | 554.72 | 554.72  (singular model) | All |
| Physical danger + humiliation | 551.52 | 551.52  (singular model) | None |
| Humiliation + entrapment | 551.05 | 551.05  (singular model) | None |
| Physical danger + entrapment | 551.63 | 551.63  (singular model) | Entrapment |
| **Entrapment** | **547.36** | 547.36  (singular model) | None |
| Humiliation | 548.43 | 531.31  (singular model) | None |

Note: All continuous predictors and covariates were standardized before running the LME models to minimize multicollinearity. Bolded=LME model with the lowest conditional AIC


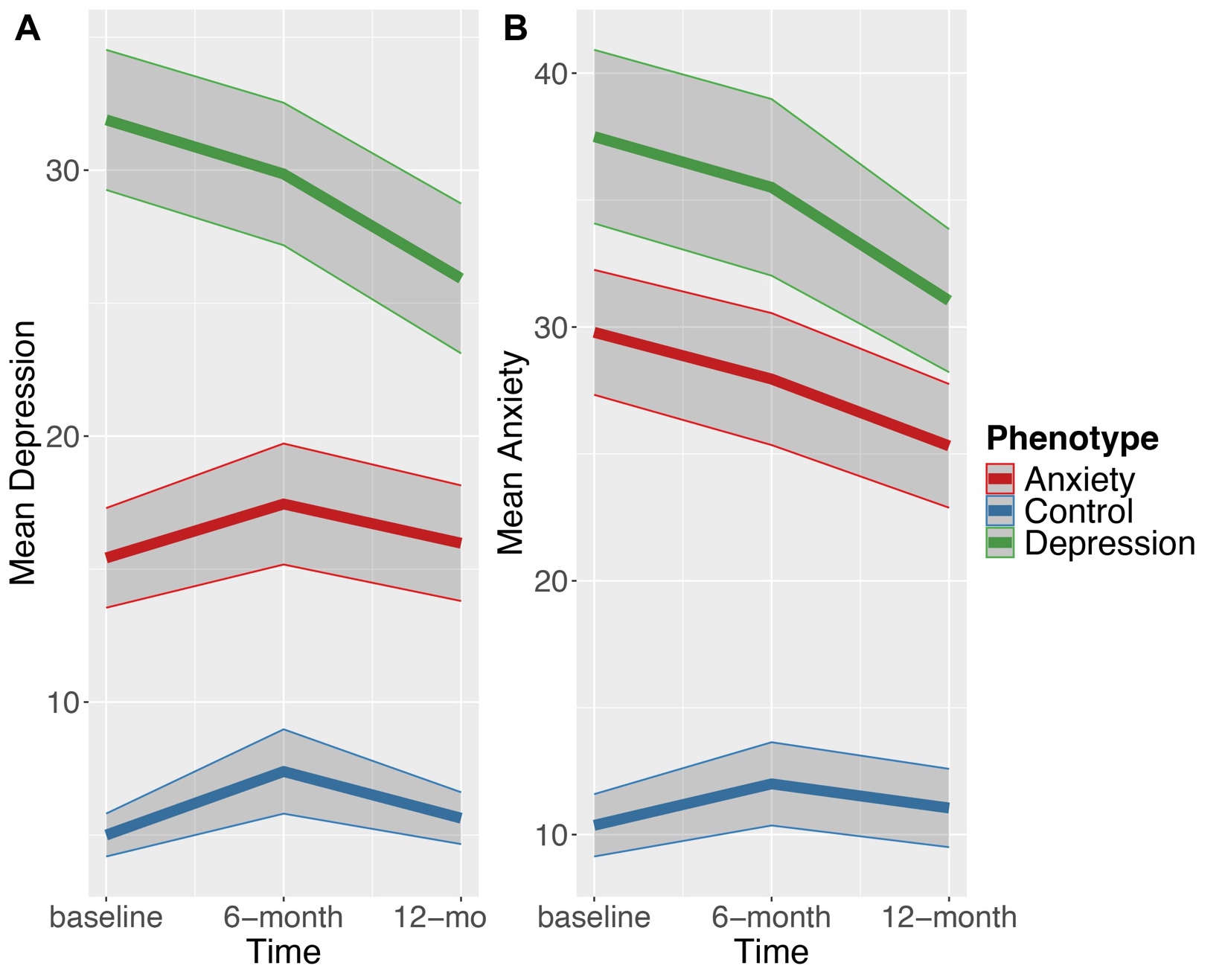


**Supplementary Figure 1.** Mean depressive and anxiety symptoms within each diagnostic group at baseline, 6-month follow-up assessment and 12-month follow-up assessment. Anxiety = having a current diagnosis of at least one anxiety disorder and no depressive disorder; Depression = having a current diagnosis of at least one depressive disorder; Control = having no current or lifetime diagnosis of any psychiatric disorder. Shades around each line represent standard error.
